# Oculist vs. Optician.—A Protest2Read at the semi-annual meeting of the Medical Society of the County of Erie, June 13, 1899.

**Published:** 1899-09

**Authors:** John J. Finerty

**Affiliations:** Buffalo, N. Y.; Oculist to the Buffalo Sisters of Charity Hospital


### OCULIST VS. OPTICIAN.—A PROTEST.[2]

By JOHN J. FINERTY, M. D., Buffalo, N. Y.
Oculist to the Buffalo Sisters of Charity Hospital.

A RECENT advertisement in the daily newspapers of an optical concern setting forth the wonderful work they were doing in the line of refraction and proclaiming the fact that many physicians were sending them patients, determined me that time for a protest was ripe. This is rather a delicate subject for an oculist to handle as his motive is liable to be misconstrued, but I think all will acknowledge the necessity of it, and I hope that after this short paper, and the discussion whch I trust it will bring out, its importance will be more fully understood.

Just as the medical profession has its quacks, the optical business has its charlatans. They pursue the same methods, and their object is

1. The report, published in the August edition of this journal, page 25, was adopted by a unanimous vote.—Editor.

2. Read at the semi-annual meeting of the Medical Society of the County of Erie, June 13, 1899.

the same: to extort money from their dupes. Judging from this advertisement at least one of these concerns has been able to use members of the medical profession as tools in their nefarious schemes. Why this is so, I am unable to answer. We can easily understand how a person possessing no knowledge of this subject can be enticed by their unwarrantably pretentious advertisements and for the sake of economy to give them a trial, but how an educated physician, who is acquainted with the wonderful anatomy and physiology of the eye, can refer his clientèle to them is beyond my comprehension. Lest I may be misunderstood, I would like to go on record here as saying, that educated opticians, as a rule, do not belong to this class. Their business is the grinding of lenses and proper adjustment of frames. These men receive and fill prescriptions of the oculist. They bear the same relation to him that the prescription druggist does to the general practitioner of medicine. The others, the so-called graduate opticians, the sight specialists, and the like, may be placed in the same class as the counter-prescribing druggist.

The fact that patients go from the general practitioner to the optician may be due to carelessness. The physician tells a patient who is suffering from headache that he ought to have his eyes examined. Left to himself to choose, he is probably not acquainted with an oculist, and goes to the optician. Such a case came under my notice not long since. A man suffered from asthenopic symptoms, had been told to have his eyes examined and went to an advertising optician, who prescribed weak concave cylinders. Upon examination I found he had no appreciable refractive error, but suffered from a weakness of the internal recti muscles. In this case the glasses, as would be expected, really intensified the symptoms. Again, this may happen from the desire of the patient to escape the fee of the oculist. This is false economy. In the majority of cases the relief, if any is obtained, is but temporary. The oculist is consulted later. He finds it necessary to change the lenses and possibly the frames. Thus the patient must purchase at least two pairs of lenses.

Quite recently I saw a case of this character in which the optician prescribed a pair of lenses which were absolutely of no use. She was a poor girl and I sent her to him with a request that he exchange her lenses for those I had prescribed. He informed her that the glasses he fitted were correct, that the lenses ordered were so different I must be mistaken, and if she wanted them she would have to pay him $2.00. She went to another optician, had the prescription filled and has since been comfortable. I mention this case to show that these merchants are not wanting in assurance.

Frequently these patients are suffering from deep-seated trouble, which can only be detected by a physician. Such a man recently consulted me wearing a pair of glasses, sold him by an optician, for the relief of headache. The cause of his trouble was a neuro-retinitis, affecting both nerves and due to syphilis. Another man came to my dispensary service suffering with an iritis, for which glasses had been prescribed. Many more cases illustrative might be cited, but these will suffice for my purpose.

These tradesmen know nothing about the diseases of the eye, as can be determined from the foregoing cases, and oftentimes commit errors which result in harm. In both of the cases referred to the eyes needed rest. In place of this they were wearing illy fitted glasses, which if anything increased the work of their eyes.

In regard to their ability to substantiate their claims, let us concisely consider what is meant by an anomaly of refraction. A normal eye is called emmetropic. Such an eye at rest is so focused that parallel rays of light striking it are brought to a point on the retina. Hypermetropia and myopia are usually axial and as such we will consider them.

A hypermetropic eye is one in which parallel rays of light are brought to a focus back of the retina. This in comparison with an emmetropic eye would be of shorter axis. The correction is made in supplying the convex glass, which will bring the focus on the retina.

The myopic eye is just the opposite of the preceding. It is a long eye, by many claimed to be pathological, and parallel rays of light are brought to a focus in front of the retina. It is corrected by the concave lens, which carries the focus back to the retina. These anomalies may or may not be complicated by astigmatism, a condition in which a point as a point cannot be brought to a focus on the retina. In the foregoing the eye is supposed to be in a state of rest—static refraction—and if it remain so correction would be an easy mechanical matter.

But there is another form of refraction, called dynamic. This is produced by the action of the ciliary muscle. It is due to the action of this muscle that we are able to accommodate the eye for near objects. It is this muscle which conceals hypermetropia and changes it frequently into apparent myopia. In the great majority of cases, especially those under forty years of age, in order to determine positively the refractive error, this muscle must be paralysed by the use of a mydriatic. I am aware some men claim that by the use of the ophthalmoscope, ophthalmometer and test-types this becomes unnecessary, but these men will have to admit that after using these tests they are uncertain as to the absolute error of refraction.

A few years ago a committee appointed by the American Ophthalmological Association reported that the best objective test for determining the refraction of the eye is the shadow test. That this will not bring out the total error without a mydriatic I know from personal experience, for I have several times observed the refraction change during mydriasis at different stages of paralysis of the accommodation.

These so-called specialists consider the eye as an optical instrument and forget that it is subject to physiological laws. They are not aware that even in moderate degrees of refractive error, while each eye may be corrected so that it has normal vision and a normal amount of convergence, thus working normally, still the coöperation of the two is found to be more or less hindered, and that accommodation and convergence are intimately connected and can be disassociated only within certain limits. On this subject Landolt says:

That however intimate the relationship between the doctrine of ocular refraction and physics, we must be careful not to consider the former from a too mathematical point of view. Although we may establish rules. exceptions to them are numerous and figures cannot often lay claim to greater authority than those of physiology in general. We shall succeed just as much better in adopting eyes to what they are destined to accomplish, in proportion as we associate more closely perfect knowledge of theory with the most careful clinical observation."

To sum up, we have in our midst men posing as expert refractionists who by their work show they are not even good mechanics. They charge extortionate prices, presumably for glasses, but in reality for their advice. They prescribe glasses when the eye is diseased and thus do a positive injury. They claim the correction of refractive anomalies is mechanical and belongs to them. I believe this work comes under the domain of ophthalmic surgery, and beg to suggest that our board of censors take this matter up and with their attorney determine whether or not these men are to continue practising an important branch of the science of medicine.

429 FRANKLIN STREET.

